# 

**Published:** 1911-09-02

**Authors:** 


### THE QUESTION BOX.

### HOME FOR EPILEPTIC BOY.

*To the Editor of* The Hospital.

Sir,—Can you or any of your readers advise me where a boy aged about seventeen years and epileptic can be placed, and where he will be kindly treated and looked after? No colony will take him as he is quite helpless and unable to do anything for himself. Moderate terms are essential.

I am, yours truly, H.H.

### THE WEEK'S NOVELTIES.

### THE DOCTOR'S WATCH.

(J. W. Benson, Ltd., 62 and 64 Ludgate Hill, E.C.)

One of the indispensable instruments in the armamentarium of every medical student and practitioner is a thoroughly reliable watch. It is needed not only as an aid, and a very important aid, to bedside examination and laboratory work, but is absolutely necessary to ensure that virtue which, although it may be merely politeness in kings, is stern duty in doctors—punctuality. The student should invest in a good watch at the commencement of his medical career, and he cannot do better than consult Messrs. J. W. Benson. This firm specialises in a "doctor's watch," which is as near the ideal as modern chronometry can attain. It is a keyless, three-quarter plate, English lever, ruby jewelled with chronometer balance, Bréguet compensation balance, and long-trotting seconds hand. The watch is listed at £12 10s. in silver, and £25 in 18-carat gold cases; while a special improved presentation quality extra large in very heavy 18-carat cases is listed at £40.

